# Estimating and testing the influence of early diagnosis on cancer survival via point effects of diagnoses and treatments

**DOI:** 10.1177/09622802221098429

**Published:** 2022-05-04

**Authors:** Xiaoqin Wang, Johannes Blom, Weimin Ye, Li Yin

**Affiliations:** 1 Department of Electrical Engineering, Mathematics, and Science, University of Gävle, Sweden; 2 Department of Clinical Science and Education, Södersjukhuset, 27106 Karolinska Institutet, Stockholm, Sweden; 3 Department of Medical Epidemiology and Biostatistics, 27106 Karolinska Institutet, Sweden

**Keywords:** Blip effect, cancer diagnosis, causal effect, G-formula, point effect, treatment regime

## Abstract

A cancer diagnosis is part of a complex stochastic process, which involves patient's characteristics, diagnosing methods, an initial assessment of cancer progression, treatments and a certain outcome of interest. To evaluate the performance of diagnoses, one needs not only a consistent estimation of the causal effect under a specified regime of diagnoses and treatments but also reliable confidence interval, *P*-value and hypothesis testing of the causal effect. In this article, we identify causal effects under various regimes of diagnoses and treatments by the point effects of diagnoses and treatments and thus are able to estimate and test these causal effects by estimating and testing point effects in the familiar framework of single-point causal inference. Specifically, using data from a Swedish prognosis study of stomach cancer, we estimate and test the causal effects on cancer survival under various regimes of diagnosing and treating hospitals including the optimal regime. We also estimate and test the modification of the causal effect by age. With its simple setting, one can readily extend the example to a large variety of settings in the area of cancer diagnosis: different personal characteristics such as family history, different diagnosing procedures such as multistage screening, and different cancer outcomes such as cancer progression.

## 1 Introduction

A cancer diagnosis is part of a complex stochastic process, in which patients’ personal and social characteristics influence the choice of diagnosing methods, diagnosing methods in turn influence the initial assessment of cancer stage, cancer stage, in turn, influences the choice of treating methods and treating methods in turn influence cancer outcomes such as cancer survival. To evaluate the performance of cancer diagnoses, one needs to estimate and test the causal effect under a specified regime of diagnoses and treatments, shorthanded as sequential causal effect (SCE). An important example of the SCE is the one under the optimal regime.

In the causal inference of treatment sequence, Robins derived the well-known G-formula, which identifies the SCE under a regime of treatments via standard parameters.^1,2^ Based on Robins’ G-formula, a parametric likelihood-based approach has been developed for estimating the SCE via standard parameters.^3,4^ If the time-dependent covariates between treatments are posttreatment variables from the previous treatments as well as confounders for the subsequent treatments, however, this approach may suffer from the curse of dimensionality and the null paradox.^3,4^ To avoid these problems, semi-parametric non-likelihood-based approaches are developed, for instance, the marginal structural model – including the doubly robust estimation – based on the inverse probability of treatment weighting^4–6^ and the G-estimation based on the structural nested mean model (SNMM) or optimal-regime SNMM.^2,4,7^ In a recent review on the optimal regime, Kosorok and Laber^8^ highlighted the need for reliable methods of evaluating the uncertainty in estimating SCEs (Section 6, statistical inference).

Recently, Wang and Yin^9,10^ derived a new version of the G-formula, which identifies the SCE by the point effects of individual treatments in the sequence. The point effect of treatment is simply the one in the causal inference of single-point treatment. Its estimation is well studied in the framework of single-point causal inference, for instance, it can be likelihood-based and further doubly robust to possible misspecification of the outcome model or the treatment assignment model.^11^ The estimation does not suffer from the curse of dimensionality and the null paradox. Based on the new G-formula, they proposed a parametric likelihood-based approach, in which they were able to estimate and test the SCE without suffering from the curse of dimensionality and the null paradox by estimating and testing the point effects. They achieved not only an unbiased estimate of the SCE but also the nominal level of coverage probability for the confidence interval.

In this article, we study the application of the parametric likelihood-based approach based on the new G-formula to the area of an early cancer diagnosis. We estimate and test SCEs under various regimes, including the optimal one, of diagnoses and treatments as well as the modifications of SCEs by covariates. We will illustrate our method by an interesting example using data from a Swedish prognosis study of stomach cancer and provide practical advice.

## 2 Data and the assumption for estimating and testing SCEs

In Sweden, patients usually seek medical help at hospitals near their residential areas (namely, catchment areas). When cancer is diagnosed, they may stay at the diagnosing hospital or transfer to another hospital for treatment. The hospital diagnosing cancer is called the diagnosing hospital, while the one treating cancer is called the treating hospital. To evaluate the performance of diagnosing and treating hospitals, we may study cancer outcomes under various regimes of diagnosing and treating hospitals among cancer patients after adjusting for patients’ differences. A question of relevance to public health policy is which type of the diagnosing and treating hospitals, large versus small, performs better on cancer survival.

The data used in this study is from a prognosis study conducted during the period between 1988 and 1995 in hospitals in central and northern Sweden.^12^ It contained information on 910 patients with stomach cancer. The large type refers to the regional or county hospitals and the small type to local hospitals. The diagnosing hospital is the treatment variable $Z_{1}$ , which takes the value $z_{1}=0$ for small type and $z_{1}=1$ for large type. The treating hospital is the treatment variable $Z_{2}$ , which takes $z_{2}=0$ for small type and $z_{2}=1$ for large type.

The following stationary covariates before $Z_{1}\;$ are measured: gender $X_{11}$ , geographic area $X_{12}$ and age $X_{13}$ . Gender is $x_{11}=0$ for females and $x_{11}=1$ for males. The geographic area is categorized into $x_{12}=0$ for rural and $x_{12}=1$ for urban. Age takes continuous values $x_{13}$ . The time-dependent covariate between $Z_{1}$ and $Z_{2}$ is cancer stage $X_{2}$ taking the values $x_{2}=1,\;2,\;3,\;4$ . The outcome of interest is *Y*, which takes y=0 for death and y=1 for survival within one year after diagnosis or within 3 years. The data and code for all analyses are available in Supplemental Data of this article. The descriptive statistics are given in Table 1.

**Table 1.** Frequencies or means (standard deviations) of covariates and outcome across the diagnosing and treating hospitals for 910 stomach cancer patients.

	Stomach cancer: frequency or mean (standard deviation) on levels of $z_{1}$ and $z_{2}$			
	$z_{1}=0$	$z_{1}=1$	$z_{2}=0$	$z_{2}=1$
N	371	539	327	583
$x_{11}=0$	153	212	137	228
=1	218	327	190	355
$x_{12}=0$	215	259	184	290
=1	156	280	143	293
$x_{13}$	71.38 (11.10)	71.76 (11.10)	71.96 (10.80)	71.41 (11.28)
$x_{2}\;=1$	56	100	49	107
=2	43	55	41	57
=3	111	117	93	135
=4	161	267	144	284
One year survival				
y=0	209	310	186	333
=1	162	229	141	250
Three-year survival				
y=0	292	413	259	446
=1	79	126	68	137

•Hospital types: $\;z_{1}=0,\;1$ for small and large diagnosing hospitals; $z_{2}=0,\;1$ for small and large treating hospitals.

•Stationary covariates: $x_{11}=0,\;1$ for female and male; $x_{12}=0,\;1$ for rural and urban residential areas; $x_{13}$ for age.

•Time-dependent covariate: $x_{2}=1,\;2,\;3,\;4$ for cancer stages.

•Outcome: y=0,1 death and survival within one year after diagnosis or within 3 years.

Due to a long-term social welfare system and relatively uniform culture in Sweden, we assume that gender $x_{11}$ , geographic area $x_{12}$ and age $x_{13}$ are the only confounders for $Z_{1}$ and additional cancer stage $x_{2}$ for $Z_{2}$ . Together with positivity and consistency assumptions, this assumption of no hidden confounders is the identifying condition.^2,3^ Under the identifying condition, we can estimate and test SCEs under various regimes of diagnosing and treating hospitals by using the observed data.

Because the cancer stage has a significant influence on diagnosing hospital as well as on treating hospital, one cannot use the standard methods such as the usual regression to estimate and test the SCE.^1,2^ In the following, we will apply the new G-formula to estimate and test the SCE via the point effects of diagnosing and treating hospitals.

## 3 Estimating and testing SCE under the regime of diagnosing and treating hospitals on one-year survival

Wang and Yin^9^ constructed point parametrization for the likelihood of all the observable variables arising from a sequence of treatments, with the point effects of treatments in the sequence as a subset of the parametrization. They showed that with this parametrization, the score functions for the point effects of treatments are little associated with one another at different treatment times, so that the point effects can be estimated separately at different treatment times, like the point effect of one single-point treatment. Furthermore, Wang and Yin^10^ derived a new version of the G-formula, which expresses the point effects in terms of the blip effects of treatments and then all other SCEs in terms of the blip effects. As a result, a likelihood approach is developed to estimating and testing the blip effect and the SCE via these point effects.^9,10^ Here, we introduce a concrete procedure of applying their method to an observational study, where the treatment assignment mechanism is unknown and the covariates can be continuous.

### 3.1 Point effects of diagnosing and treating hospitals

Let $\mu (x_{11},x_{12},x_{13},z_{1})=E(Y|x_{11},x_{12},x_{13},z_{1})$ be the mean of observable survival *Y* in the stratum $\left(x_{11},x_{12},x_{13},z_{1}\right)$ . In the causal inference of single-point treatment, the point effect of diagnosing hospital $z_{1}$ in a stratum $\left(x_{11},x_{12},x_{13}\right)$ is

$$\vartheta (x_{11},x_{12},x_{13};\;z_{1})=\mu (x_{11},x_{12},x_{13},z_{1})-\mu (x_{11},x_{12},x_{13},0)$$

This point effect is a mixed effect of $z_{1}$ and $z_{2}$ , but it will be used to obtain SCEs below. Let $\mu (x_{11},x_{12},x_{13},z_{1},x_{2},z_{2})=E(Y|x_{11},x_{12},x_{13},z_{1},x_{2},z_{2})$ be the mean of observed survival *Y* in the stratum $\left(x_{11},x_{12},x_{13},z_{1},x_{2},z_{2}\right)$ . Then the point effect of treating hospital $z_{2}$ in a stratum $\left(x_{11},x_{12},x_{13},z_{1},x_{2}\right)$ is

$$\vartheta (x_{11},x_{12},x_{13},z_{1},x_{2};\;z_{2})=\mu (x_{11},x_{12},x_{13},z_{1},x_{2},z_{2})-\mu (x_{11},x_{12},x_{13},z_{1},x_{2},0)$$

For a small sample, it would be efficient to use the logistic model and the Bernoulli-based distribution to estimate the point effect for a dichotomous outcome. For a large sample like ours, one may also use the linear model and the normal distribution for the estimation. For the simplicity of explication, we use linear model and normal distribution to estimate the point effect as follows.

To estimate the point effect of diagnosing hospital $z_{1}$ , we model the mean $\mu (x_{11},x_{12},x_{13},z_{1})$ , in which diagnosing hospital $z_{1}$ is the variable of interest while all others are possible confounders of $z_{1}$ . Notably, the model does not contain any posttreatment variable after $z_{1}$ , so the null paradox does not necessarily occur and the model can be unsaturated without biasing the point effect. At the significance level of 0.10, the residential area $x_{12}$ is not significant, in line of the medical observation that the residential area is not as influential as gender and age. So we remove it from the model. Furthermore, the interactions between $z_{1}$ and $\left(x_{11},x_{12},x_{13}\right)$ are not significant either. Finally, we obtain

(1) $$\mu (x_{11},x_{13},z_{1})=\beta_{1}+\beta_{2}x_{11}+\beta_{3}x_{13}+\vartheta_{1}z_{1}$$

From this model, we obtain the estimate for the point effect of $z_{1}=1$ , that is, $\vartheta (x_{11},x_{13};\;1)=\mu (x_{11},x_{13},1)-\mu (x_{11},x_{13},0)=\vartheta_{1}$ , which is the same for all $\left(x_{11},x_{13}\right)$ . The estimate ${\hat{\vartheta }}_{1}$ is presented in Table 2.

**Table 2.** Point effects, blip effects and optimal SCEs of diagnosing and treating hospitals on one-year survival of stomach cancer: estimate, *P*-value and 95% CI.

	Estimate, *P*-value and 95% CI in column for various causal effects				
	$\vartheta_{1}$	$\vartheta_{21}$	$\vartheta_{22}$	$\vartheta_{23}$	$\vartheta_{24}$
Point	−0.007	0.005	0.089	0.027	−0.034
effect	0.819	0.918	0.357	0.682	0.382
	(−0.071,0.056)	(−0.083,0.092)	(−0.100,0.277)	(−0.103,0.158)	(−0.109,0.042)
	$\gamma_{1}$	$\gamma_{21}$	$\gamma_{22}$	$\gamma_{23}$	$\gamma_{24}$
Blip	−0.009	0.005	0.089	0.027	−0.034
effect	0.710	0.918	0.357	0.682	*0.382*
	(−0.054,0.037)	(−0.083,0.092)	(−0.100,0.277)	(−0.103,0.158)	(−0.109,0.042)
	$\Gamma_{1}$	$\Gamma_{21}$	$\Gamma_{22}$	$\Gamma_{23}$	$\gamma_{24}$
Optimal	0.028	0.053	0.089	0.027	−0.034
SCE	0.237	0.220	0.357	0.682	0.382
	(−0.019,0.075)	(−0.032,0.138)	(−0.100,0.277)	(−0.103,0.158)	(−0.109,0.042)
Estimate, *P*-value and 95% CI for effect modification of large diagnosing hospital by age: $\gamma_{1,2}$ : − 0.001, 0.298, (−0.003,0.001) ; for intercept: $\gamma_{1,1}$ : 0.064, 0.378, (−0.078,0.205) .					
Estimate, *P*-value and 95% CI for effect modification of large treating hospital by age: $\gamma_{21,2}$ : 0.012, 0.037, (0.001,0.023) ; for intercept: $\gamma_{21,1}$ : − 0.801, 0.030, (−1.526,−0.077) .					

• Five point effects: $\vartheta_{1}$ of large versus small diagnosing hospital; $\vartheta_{2j}$ of large versus small treating hospital for cancer stage j=1,2,3,4.

• Five blip effects: $\gamma_{1}$ of large versus small diagnosing hospital; $\gamma_{2j}$ of large versus small treating hospital for cancer stage j=1,2,3,4.

• Five optimal SCEs: $\Gamma_{1}$ under optimal regime of diagnosing and treating hospital; $\Gamma_{2j}$ of optimal treating hospital for cancer stage j=1,2,3; $\Gamma_{24}=0$ of optimal treating hospital for cancer stage 4 is the baseline, so the blip effect $\;\gamma_{24}$ is instead presented.

• Modification of the blip effect by age: $\gamma_{1,2}$ modifying the blip effect of large diagnosing hospital by age; $\gamma_{1,1}$ the intercept of the blip effect; $\gamma_{21,2}$ modifying the blip effect of large treating hospital for cancer stage 1 by age; $\gamma_{21,1}$ the intercept for the blip effect.

To estimate the point effect of treating hospital $z_{2}$ , we model the mean $\mu (x_{11},x_{12},x_{13},z_{1},x_{2},z_{2})$ , in which treating hospital $z_{2}$ is the variable of interest while all others are possible confounders of $z_{2}$ . Notably, the model does not contain any posttreatment variable after $z_{2}$ , so the null paradox does not necessarily occur and the model can be unsaturated without biasing the point effect. At the significance level of 0.10, residential area $x_{12}$ and diagnosing hospital $z_{1}$ are not significant, in line of the medical observations that (a) residential area is not as influential as gender $x_{11}$ and age $x_{13}$ and (b) the diagnosing hospital influences cancer outcomes only via cancer stages $x_{2}$ diagnosed. Furthermore, patients with different cancer stages $x_{2}=1,\;2,\;3,\;4$ usually have rather different survivals, and so we model the mean $\mu (x_{11},x_{12},x_{13},z_{1},x_{2},z_{2})$ separately for different cancer stages. Finally, we obtain

(2) $$\begin{array}{c} \mu (x_{11},x_{13},x_{2}=1,z_{2})=\beta_{4}+\beta_{5}x_{11}+\beta_{6}x_{13}+\vartheta_{21,1}z_{2}+\vartheta_{21,2}z_{2}x_{13}\\ \mu (x_{2}=2,z_{2})=\beta_{7}+\vartheta_{22}z_{2}\\ \begin{array}{c} \mu (x_{2}=3,z_{2})=\beta_{8}+\vartheta_{23}z_{2}\\ \mu (x_{2}=4,z_{2})=\beta_{9}+\vartheta_{24}z_{2} \end{array} \end{array}$$

In the sub models for cancer stage $x_{2}=2,\;3,\;4$ , gender $x_{11}$ and age $x_{13}$ are not significant in line with the medical observation that gender and age have less influence on cancer survival with advanced stages. For the cancer stage $x_{2}=1,$ the point effect of $z_{2}=1$ in stratum $\left(x_{11},x_{13}\right)$ is $\vartheta (x_{11},x_{13},x_{2}=1;\;1)=\mu (x_{11},x_{13},x_{2}=1,\;1)-\mu (x_{11},x_{13},x_{2}=1,\;0)=\vartheta_{21,1}+\vartheta_{21,2}x_{13}$ . Its average over $\left(x_{11},x_{13}\right)$ is the point effect of $z_{2}=1$ for cancer stage $x_{2}=1$ and is equal to $\vartheta_{21,1}+\vartheta_{21,2}E(x_{13}|x_{2}=1)$ , denoted by $\vartheta_{21}.$ For cancer stages $x_{2}=2,\;3,\;4$ , the point effects of $z_{2}=1$ are $\vartheta (x_{2};\;1)=\mu (x_{2},1)-\mu (x_{2},0)$ , which are equal to $\vartheta_{22},\;\vartheta_{23},\;\vartheta_{24}$ , respectively. The estimates ${\hat{\vartheta }}_{21},$ ${\hat{\vartheta }}_{22}$ , ${\hat{\theta }}_{23}$ and ${\hat{\vartheta }}_{24}\;$ are presented in Table 2.

Briefly, we estimate the point effects of diagnosing and treating hospitals by modelling the means $\mu (x_{11},x_{12},x_{13},z_{1})$ and $\mu (x_{11},x_{12},x_{13},z_{1},x_{2},z_{2})$ in the usual framework of regression.

### 3.2 Blip effects of diagnosing and treating hospitals

Because neither (1) nor (2) contains the geographic area $x_{12}$ , we do not include $x_{12}$ in the following development. Here, we consider a special type of SCEs, called the blip effects, from which all other SCEs are determined,^9,10^ see also the next subsection. The blip effect $\phi (x_{11},x_{13};\;z_{1})$ of diagnosing hospital $z_{1}$ in the stratum $\left(x_{11},x_{13}\right)$ is an increase of the mean of potential survival when potentially and deterministically assigning a regime of diagnosing hospital $z_{1}$ and small treating hospital versus a regime of small diagnosing hospital and small treating hospital to the stratum. Under the identifying condition, Robins’ G-formula expresses the blip effect of $z_{1}$ in terms of the means $\mu (x_{11},x_{13},z_{1},x_{2},z_{2})$ by^1,2^

$$\phi (x_{11},x_{13};\;z_{1})=E\{\mu (x_{11},x_{13},z_{1},x_{2},0)|x_{11},x_{13},z_{1}\}-E\{\mu (x_{11},x_{13},0,x_{2},0)|x_{11},x_{13},0\}$$

where the expectations are with respect to $P(x_{2}|x_{11},x_{13},z_{1})$ for $z_{1}$ and $z_{1}=0$ . Clearly, the blip effect of small diagnosing hospital $\phi (x_{11},x_{13};0)=0$ . The blip effect $\phi (x_{11},x_{13},z_{1},x_{2};\;z_{2})$ of treating hospital $z_{2}$ in the stratum $\left(x_{11},x_{13},z_{1},x_{2}\right)$ is an increase of the mean of potential survival when potentially and deterministically assigning treating hospital $z_{2}$ versus 0 to the stratum. Under the identifying condition, Robins’ G-formula gives^1,2^

$$\phi (x_{11},x_{13},z_{1},x_{2};\;z_{2})=\mu (x_{11},x_{13},z_{1},x_{2},z_{2})-\mu (x_{11},x_{13},z_{1},x_{2},0)$$

Clearly, the blip effect of small treating hospital $\phi (x_{11},x_{13},z_{1},x_{2};\;0)=0$ . Based on Robins’ G-formula, one can estimate $\phi (x_{11},x_{13};\;z_{1})$ and $\phi (x_{11},x_{13},z_{1},x_{2};\;z_{2})$ by modelling $\mu (x_{11},x_{13},z_{1},x_{2},z_{2})$ .^3,4^ However, in the model, both $z_{1}$ and $z_{2}$ are the variables of interest while $x_{2}$ is a posttreatment variable of $z_{1}$ and a confounder of $z_{2}$ , so the modelling suffers from the curse of dimensionality and the null paradox.

An interesting clinical observation in cancer diagnosis is that young patients diagnosed at small hospitals tend to have poor prognoses.^13^ This phenomenon is known as doctors’ delay, but little-studied statistically. Motivated by this clinical observation, we suppose that $\phi (x_{11},x_{13};\;1)$ is a linear function of age $x_{13},$ that is, age is a modifier of the blip effect. We also studied gender $x_{11}$ as an effect modifier, but found that the effect modification by gender was not significant at all. Further motivated by the point effect $\vartheta (x_{11},x_{13},x_{2};z_{2})$ obtained in Section 3.1, where $z_{2}$ is noticeably the last treatment variable, we suppose that the blip effects satisfy SNMM of the form

(3) $$\begin{array}{c} \phi (x_{11},x_{13};\;z_{1})=\gamma_{1,1}z_{1}+\gamma_{1,2}x_{13}z_{1}\\ \phi (x_{11},x_{13},z_{1},x_{2}=1;\;z_{2})=\gamma_{21,1}z_{2}+\gamma_{21,2}x_{13}z_{2}\\ \begin{array}{c} \phi (x_{11},x_{13},z_{1},x_{2}=2;\;z_{2})=\gamma_{22}z_{2}\\ \begin{array}{c} \phi (x_{11},x_{13},z_{1},x_{2}=3;\;z_{2})=\gamma_{23}z_{2}\\ \phi (x_{11},x_{13},z_{1},x_{2}=4;\;z_{2})=\gamma_{24}z_{2} \end{array} \end{array} \end{array}$$

Let $\gamma =(\gamma_{1,1},\;\gamma_{1,2},\gamma_{21,1},\;\gamma_{21,2},\gamma_{22},\gamma_{23},\gamma_{24})$ be the vector of all parameters in (3). The average of the blip effect $\phi (x_{11},x_{13};1)$ of $z_{1}=1\;$ over $\left(x_{11},x_{13}\right)$ is equal to $\gamma_{1,1}+\gamma_{1,2}E(x_{13})$ , denoted by $\gamma_{1}$ . Then $\gamma_{1}$ $=\gamma_{1,1}+\gamma_{1,2}E(x_{13})$ is the blip effect of $z_{1}=1$ in the population. The parameter $\gamma_{1,2}$ is the modification of $\gamma_{1}$ by $x_{13}$ (age). The average of the blip effect $\phi (x_{11},x_{13},z_{1},x_{2}=1;\;1)$ of $z_{2}=1\;$ over $\left(x_{11},x_{13},z_{1}\right)$ is equal to $\gamma_{21,1}+\gamma_{21,2}E(x_{13}|x_{2}=1)$ , denoted by $\gamma_{21}$ . Then $\gamma_{21}=\gamma_{21,1}+\gamma_{21,2}E(x_{13}|x_{2}=1)$ is the blip effect of $z_{2}=1\;$ for cancer stage $x_{2}=1.$ The parameter $\gamma_{21,2}$ is the modification of $\gamma_{21}$ by $x_{13}$ .

Now by applying the new G-formula^10^ (see also formula (10) for t=1 with T=2 in Supplemental Data), we have

$$\begin{array}{rl} \vartheta (x_{11},x_{13};\;1)= & \phi (x_{11},x_{13};\;1)\\ & \;+E\{\phi (x_{11},x_{13},z_{1},x_{2};\;z_{2})|x_{11},x_{13},z_{1}=1\}-E\{\phi (x_{11},x_{13},0,x_{2};\;z_{2})|x_{11},x_{13},z_{1}=0\} \end{array}$$

where the expectation is with respect to $P(x_{2},\;z_{2}|x_{11},x_{13},z_{1})$ for $z_{1}=1$ and $z_{1}=0.$ This formula shows that $\vartheta (x_{11},x_{13};1)$ decomposes into the blip effects of $z_{1}=1$ and $z_{2}.$ Noticing that $\phi (x_{11},x_{13},z_{1},x_{2};\;0)=0$ , we have

$$E\{\phi (x_{11},x_{13},z_{1},x_{2};\;z_{2})|x_{11},x_{13},z_{1}\}\;=\overset{4}{\underset{\;j=1}{\sum }}\;\phi (x_{11},x_{13},z_{1},x_{2}=j;\;1)P(x_{2}=j,\;z_{2}=1|x_{11},x_{13},z_{1})$$

Let $C_{j}(x_{11},x_{13})=P(x_{2}=j,\;z_{2}=1|x_{11},x_{13},\;z_{1}=1)-P(x_{2}=j,\;z_{2}=1|x_{11},x_{13},\;z_{1}=0),\;\;j=1,\;2,\;3,\;4$ , be the probability differences. Then under SNMM (3), we have

(4) $$\vartheta (x_{11},x_{13};\;1)\;=\gamma_{1,1}+\gamma_{1,2}x_{13}+(\gamma_{21,1}+\gamma_{21,2}x_{13})C_{1}(x_{11},x_{13})+\overset{4}{\underset{\;j=2}{\sum }}\;\gamma_{2j}C_{j}(x_{11},x_{13})$$

The point effect $\vartheta (x_{11},x_{13};1)$ has been estimated from model (1) in Section 3.1. The probability differences $C_{j}(x_{11},x_{13})$ are estimated by the observed proportion differences ${\hat{C}}_{j}(x_{11},x_{13})$ in the data without modelling.

Because $Z_{2}$ is the last treatment variable, we have $\vartheta (x_{11},x_{13},x_{2};\;1)=\phi (x_{11},x_{13},z_{1},x_{2};\;1),$ where the point effect $\vartheta (x_{11},x_{13},x_{2};\;1)$ has been estimated from model (2) in Section 3.1. Therefore, under SNMM (3), we have

(5a) $$\begin{array}{c} \vartheta_{21,1}=\gamma_{21,1}\\ \vartheta_{21,2}=\gamma_{21,2}\\ \begin{array}{c} \vartheta_{22}=\gamma_{22}\\ \vartheta_{23}=\gamma_{23}\\ \vartheta_{24}=\gamma_{24} \end{array} \end{array}$$

From the estimates of the point effects, we can estimate the blip effects by applying (4) and (5a).^10^ The point effects are not necessarily biased, and neither are those of the blip effects if SNMM (3) is not misspecified.^10^

Because age $x_{13}\;$ is continuous and the number of its values may be large, it is difficult to directly use formula (4) to estimate the blip effects. On the other hand, the blip effects $\phi (x_{11},x_{13};\;1)$ are indexed by only two parameters $\gamma_{1,1}$ and $\gamma_{1,2}$ under SNMM (3). Hence, we may use the standardized point effects of $z_{1}=1$ on a small number of subpopulations of patients at the time of diagnosis to estimate the blip effect.

As an illustration, let $S_{1}=L$ be the subpopulation with patients’ age ( $x_{13}$ ) lower than the median age at the time of diagnosis and $S_{1}=H$ with age higher than or equal to the median age. Then, we have two standardized point effects of $z_{1}=1,$ which are $\Theta (S_{1};\;1)=E(\vartheta (x_{11},x_{13};\;1)|S_{1})$ with respect to the probability $P(x_{11},x_{13}|S_{1})$ for $S_{1}=L,\;\;H$ . This procedure is known as the standardization in Epidemiology.

Let $C_{j}(S_{1})=E\{C_{j}(x_{11},x_{13})|S_{1}\}$ ( j=1,2,3,4) be the mean of $C_{j}(x_{11},x_{13})$ with respect to $P(x_{11},x_{13}|S_{1})$ . Then by taking the average of formula (4) with respect to $P(x_{11},x_{13}|S_{1})$ , we obtain

(5b) $$\Theta (S_{1};\;1)=\gamma_{1,1}+\gamma_{1,2}E(x_{13}|S_{1})+\gamma_{21,1}C_{1}(S_{1})+\gamma_{21,2}E\{x_{13}C_{1}(x_{11},x_{13})|S_{1}\}+\overset{4}{\underset{\;j=2}{\sum }}\;\gamma_{2j}C_{j}(S_{1}),\;\;\;\;S_{1}=L,H$$

The estimates $\hat{E}(x_{13}|S_{1}),$ $\hat{E}\{x_{13}C_{1}(x_{11},x_{13})|S_{1}\}$ , ${\hat{C}}_{j}(S_{1})$ ( j=1,2,3,4) are obtained, respectively, by averaging $x_{13}$ with respect to $\hat{P}(x_{13}|S_{1}),$ $x_{13}{\hat{C}}_{j}(x_{11},x_{13})$ with respect to $\hat{P}(x_{11},\;\;x_{13}|S_{1}),$ and ${\hat{C}}_{j}(x_{11},\;\;x_{13})$ with respect to $\hat{P}(x_{11},\;\;x_{13}|S_{1})$ , where all proportions involved are observed from data, not from modelling. Based on model (1) in the previous subsection, we have $\vartheta (x_{11},x_{13};\;1)=\vartheta_{1}$ and thus $\Theta (S_{1};\;1)=E\{\vartheta (x_{11},x_{13};\;1)|S_{1}\}=\vartheta_{1}$ . Therefore, we have $\hat{\Theta }(S_{1};\;1)={\hat{\vartheta }}_{1}$ , but the variance $\mathrm{var}\{\hat{\Theta }(S_{1};\;1)\}$ is obtained by adjusting $\mathrm{var}({\hat{\vartheta }}_{1})$ to the size of subpopulation $S_{1}$ .

Now, conditional on all covariates, diagnosing and treating hospitals, we use (5a) and (5b) as a regression model to estimate $\gamma =(\gamma_{1,1}$ , $\gamma_{1,2}$ , $\gamma_{21,1}$ , $\gamma_{21,2}$ , $\gamma_{22}$ , $\gamma_{23},$ $\gamma_{24})$ , where the response variables are the estimates $\hat{\Theta }(S_{1};\;1)$ , ${\hat{\vartheta }}_{21,1}$ , ${\hat{\vartheta }}_{21,2}$ , ${\hat{\vartheta }}_{2j}$ ( j=2,3,4 ) while the explanatory variables are the proportions $\hat{E}(x_{13}|S_{1}),\;$ $\hat{E}\{x_{13}C_{1}(x_{11},x_{13})|S_{1}\}$ and ${\hat{C}}_{j}(S_{1})$ ( j=1,2,3,4) and the one. These explanatory variables are obtained without modelling as describe above. The estimates $\hat{\Theta }(S_{1};\;1)={\hat{\vartheta }}_{1}$ , ${\hat{\vartheta }}_{21,1}$ , ${\hat{\vartheta }}_{21,2}$ , ${\hat{\vartheta }}_{2j}$ ( j=2,3,4 ) are obtained in Section 3.1 from models (1) and (2), but their conditional variances are obtained from the same models by specifying a constant dispersion parameter for the distribution. The bootstrap method is used to obtain the covariance matrix $\mathrm{cov}(\hat{\gamma })$ incorporating the variability of all covariates and diagnosing and treating hospitals. With $\hat{\gamma }$ and $\mathrm{cov}(\hat{\gamma })$ , we conduct the Wald test on γ .

Briefly, we estimate and test the blip effects of diagnosing and treating hospitals in the usual framework of regression. The result is presented in Table 2.

### 3.3 SCE under any regime of diagnosing and treating hospitals

First, we consider the SCE of treatment regime $D_{2}$ in the stratum $\left(x_{11},x_{13},z_{1},x_{2}\right)$ : $\mathrm{SCE}(x_{11},x_{13},z_{1},x_{2};\;D_{2})$ , which is an increase of the mean of potential survival when the regime $D_{2}$ potentially and deterministically assigned treating hospital $z_{2}$ versus 0 to the stratum . Noticeably, the regime $D_{2}$ can be dynamic, for instance, $D_{2}=1$ for patients younger than 69 years and $D_{2}=0$ otherwise. According to the new G-formula^10^ (see also formula (11) for time t=T=2 in Supplemental Data), we have $\mathrm{SCE}(x_{11},x_{13},z_{1},x_{2};\;D_{2})=\phi (x_{11},x_{13},z_{1},x_{2};z_{2}).$ Then we can readily use $\phi (x_{11},x_{13},z_{1},x_{2};z_{2})$ in SNMM (3) to obtain the following formula for the SCE under SNMM (3)

(6) $$\mathrm{SCE}(x_{11},x_{13},z_{1},x_{2};\;D_{2})=\begin{array}{c} \gamma_{21,1}z_{2}+\gamma_{21,2}x_{13}z_{2},\;\;\;\;x_{2}=1\;\;\;\;\\ \gamma_{22}z_{2},\;\;\;\;\;\;\;x_{2}=2\;\;\;\;\;\;\;\;\;\;\;\;\;\;\;\;\;\;\;\;\;\;\;\;\;\;\\ \begin{array}{c} \gamma_{23}z_{2},\;\;\;\;\;\;\;x_{2}=3\;\;\;\;\;\;\;\;\;\;\;\;\;\;\;\;\;\;\;\;\;\;\;\;\;\;\\ \gamma_{24}z_{2},\;\;\;\;\;\;\;x_{2}=4\;\;\;\;\;\;\;\;\;\;\;\;\;\;\;\;\;\;\;\;\;\;\;\;\;\; \end{array} \end{array}$$

Second, we consider the SCE of treatment regime $\left(D_{1},\;D_{2}\right)$ in the stratum $\left(x_{11},x_{13}\right)$ : $\mathrm{SCE}(x_{11},x_{13};D_{1},D_{2})$ , which is an increase in the mean of potential survival when the regime $\left(D_{1},D_{2}\right)$ potentially and deterministically assigned $\left(z_{1},\;\;z_{2}\right)$ of diagnosing and treating hospitals versus (0,0) to the stratum. According to the new G-formula^10^ (see also formula (11) for time t=1 with T=2 in Supplemental data), we have

$$\mathrm{SCE}(x_{11},x_{13};\;D_{1},D_{2})=\phi (x_{11},x_{13};\;z_{1})+E\{\phi (x_{11},x_{13},z_{1},x_{2};\;z_{2})|x_{11},x_{13},z_{1}\}$$

where the expectation is with respect to $P(x_{2}=j|x_{11},x_{13},z_{1}).$ This formula shows that the SCE decomposes into the blip effects of $z_{1}$ and $z_{2}$ in the regime. Under SNMM (3), we have

(7) $$\mathrm{SCE}(x_{11},x_{13};\;D_{1},D_{2})=\gamma_{1,1}z_{1}+\gamma_{1,2}x_{13}z_{1}$$

$$+\;(\gamma_{21,1}z_{2}+\gamma_{21,2}x_{13}z_{2})P(x_{2}=1|x_{11},x_{13},z_{1})+\underset{\;j=2,3,4}{\sum }\;\gamma_{2j}z_{2}P(x_{2}=j|x_{11},x_{13},z_{1})$$

Formulas (6) and (7) imply that the SCEs are determined by blip effects.^9,10^ Taking the average of (7) with respect to $P(x_{11},x_{13})$ , we obtain the formula for $\mathrm{SCE}(D_{1},D_{2})$ , which is an increase of the mean of the potential survival when $\left(D_{1},D_{2}\right)$ potentially and deterministically assigned $\left(z_{1},\;\;z_{2}\right)$ of diagnosing and treating hospitals versus (0,0) to the population. In (7), the probability $P(x_{2}|x_{11},x_{13},z_{1})$ is modelled in the framework of single-point causal inference by assuming a parametric model and the multinomial distribution; see also a description in Supplemental Data for a general sequence of cancer diagnoses and treatments.

In (6) replacing $\gamma =(\gamma_{1,1}$ , $\gamma_{1,2}$ , $\gamma_{21,1}$ , $\gamma_{21,2}$ , $\gamma_{22}$ , $\gamma_{23,}$ $\gamma_{24})$ by estimates $\;\hat{\gamma }$ obtained from (5a) and (5b), we obtain the estimate for $\mathrm{SCE}(x_{11},x_{13},z_{1},x_{2};\;D_{2})$ under any $D_{2}$ . In (7) replacing probability $P(x_{2}|x_{11},x_{13},z_{1})$ by proportion $\hat{P}(x_{2}|x_{11},x_{13},z_{1})$ besides γ , we obtain the estimate for $\mathrm{SCE}(x_{11},x_{13};\;D_{1},D_{2})$ under any regime $\left(D_{1},D_{2}\right)$ . Averaging $\hat{SCE}(x_{11},x_{13};\;D_{1},D_{2})$ over the proportion $\hat{P}(x_{11},x_{13})$ , we obtain the estimate for $\mathrm{SCE}(D_{1},D_{2})$ . The variance of the estimated SCE is obtained by the bootstrap method. With the estimate and its variance, we obtain the confidence interval of the SCE. The covariance between the estimated SCEs under different regimes is also obtained by the bootstrap method. With the estimates and their covariance, we conduct the Wald test on the SCEs and so compare the underlying regimes.

By the dynamic programming procedure, we estimate the optimal regime $\left(O_{1},O_{2}\right)$ of diagnosing and treating hospitals by estimating and testing SCEs, as follows. Both $\;D_{1}$ and $D_{2}$ take values 0 or 1. From the estimates $\hat{SCE}(x_{11},x_{13},z_{1},x_{2};\;D_{2})$ , we obtain ${\hat{O}}_{2}=\arg\;\mathrm{ma}x_{D_{2}}\hat{SCE}(x_{11},x_{13},z_{1},x_{2};\;D_{2})$ , which is the treating hospital such that $\hat{SCE}(x_{11},x_{13},z_{1},x_{2};\;{\hat{O}}_{2})$ achieves the maximum value of $\hat{SCE}(x_{11},x_{13},z_{1},x_{2};\;D_{2})$ for all possible $D_{2}$ ( 0 or 1) given $\left(x_{11},x_{13},z_{1},x_{2}\right)$ . For the cancer stage $x_{2}=1$ , we have according to (6)

$$\hat{SCE}(x_{11},x_{13},z_{1},x_{2}=1;\;D_{2})={\hat{\gamma }}_{21,1}z_{2}+{\hat{\gamma }}_{21,2}x_{13}z_{2}$$

which, by using ${\hat{\gamma }}_{21,1}=-0.801$ and ${\hat{\gamma }}_{21,2}=0.012$ in Table 2, achieves the maximum value when $z_{2}=1\;$ for age $x_{13}\geq 69$ years and when $z_{2}=0$ for age $x_{13}<69$ . Thus, we have ${\hat{O}}_{2}=1$ (large treating hospital) for $x_{13}\geq 69$ years and ${\hat{O}}_{2}=0$ (small treating hospital) otherwise. Here we see that ${\hat{O}}_{2}$ is a dynamic regime. Let $\;\Gamma_{21}$ be the average of $SCE(x_{11},x_{13},z_{1},x_{2}=1;{\hat{O}}_{2})$ over $x_{13}$ . For cancer stages $x_{2}=2,\;3$ , similarly, by applying (6) to the estimates ${\hat{\gamma }}_{22}=0.089\;$ and ${\hat{\gamma }}_{23}=0.027$ in Table 2, we find that ${\hat{O}}_{2}=1$ . Let $\Gamma_{22}=$ $SCE(x_{11},x_{13},z_{1},x_{2}=2;{\hat{O}}_{2})$ and $\Gamma_{23}=SCE(x_{11},x_{13},z_{1},x_{2}=3;{\hat{O}}_{2})$ . For the cancer stage $x_{2}=4$ with ${\hat{\gamma }}_{24}=-0.034$ in Table 2, we have ${\hat{O}}_{2}=0$ . Let $\Gamma_{24}=SCE(x_{11},x_{13},z_{1},x_{2}=4;{\hat{O}}_{2})$ , which is the baseline and equal to zero. Instead of $\Gamma_{24}=0$ , we present the estimate of $\gamma_{24}$ in Table 2 together with the estimates of $\Gamma_{21}$ , $\Gamma_{22}$ and $\Gamma_{23}.$

From the estimate $\hat{SCE}(x_{11},x_{13};\;D_{1},{\hat{O}}_{2})$ , we obtain ${\hat{O}}_{1}=\arg\;\mathrm{ma}x_{D_{1}}\hat{SCE}(x_{11},x_{13};\;D_{1},{\hat{O}}_{2})$ , which is the diagnosing hospital such that $\hat{SCE}(x_{11},x_{13};\;{\hat{O}}_{1},{\hat{O}}_{2})$ is the maximum value of $\hat{SCE}(x_{11},x_{13};\;D_{1},{\hat{O}}_{2})\;$ for all possible $D_{1}$ ( 0 or 1) given $\left(x_{11},x_{13}\right)$ . Specifically, we replace $D_{2}$ by ${\hat{O}}_{2}$ and γ by its estimate $\hat{\gamma }$ in (7) to get $\hat{SCE}(x_{11},x_{13};\;D_{1},{\hat{O}}_{2})$ and find that it achieves the maximum value when $z_{1}=1$ for age $x_{13}<55$ years and when $z_{1}=0$ for $x_{13}\geq 55$ . Thus, we have ${\hat{O}}_{1}=1$ (large diagnosing hospital) for $x_{13}<55$ and ${\hat{O}}_{1}=0$ (small diagnosing hospital) for $x_{13}\geq 55$ . Averaging $\hat{SCE}(x_{11},x_{13};\;{\hat{O}}_{1},{\hat{O}}_{2})$ over $\left(x_{11},x_{13}\right)$ , we obtain the estimate for $\Gamma_{1}=SCE({\hat{O}}_{1},{\hat{O}}_{2})$ , which is presented in Table 2.

Briefly, we use one estimated SNMM to estimate SCEs under various regimes and thus can compare these regimes by the hypothesis testing of the SCEs under these regimes.

### 3.4 Causal analysis of diagnosing and treating hospitals based on Table 2

First, we analyse treating hospital $z_{2}$ . For the cancer stage $x_{2}=1$ , the blip effect $\gamma_{21}$ is estimated at 0.007 with *P*-value  =  0.918, implying no overall difference between large and small treating hospitals. However, there is an effect modification by age: $\gamma_{21,2}$ estimated at 0.012 with *P*-value  =  0.037. The estimated optimal treating hospital is the small one ${\hat{O}}_{2}=0$ for age < 69 years and the large one ${\hat{O}}_{2}=1$ for age ≥ 69 years. The corresponding optimal SCE, $\Gamma_{21}$ , is estimated at 0.053 with *P*-value  =  0.220. This observation reflects the fact that old patients usually have more comorbidities and large treating hospitals are probably better in dealing with comorbidities.

For the cancer stage $x_{2}=2$ , the blip effect $\gamma_{22}$ is estimated at 0.089 with *P*-value  =  0.357, implying that a large treating hospital is better for patients with this cancer stage, albeit with somewhat small significance due to our small sample ( n=98 ). There is no effect modification by age. The estimated optimal treating hospital is the large one ( ${\hat{O}}_{2}=1)$ and the optimal SCE is equal to the blip effect: $\Gamma_{22}=\gamma_{22}$ . Similar observations are made for $x_{2}=3$ .

For the cancer stage $x_{2}=4$ , the blip effect $\gamma_{24}$ is estimated at −_ _0.034 with *P*-value  =  0.382, implying that a small treating hospital is better for patients with an advanced cancer stage, albeit with somewhat small significance. There is no effect modification by age. The estimated optimal treating hospital is the small one ( ${\hat{O}}_{2}=0)$ and the optimal SCE is equal to the baseline $\Gamma_{24}=0$ . This reflects the fact that palliative care is more central in patients with advanced stages of stomach cancer.

Second, we analyse the diagnosing hospital. The blip effect $\gamma_{1}\;$ is estimated at − 0.009 with *P*-value  =  0.710, implying no overall difference between large and small diagnosing hospitals. However, there is an effect modification by age: $\gamma_{1,2}$ estimated at − 0.001 with *P*-value  =  0.298. The estimated optimal diagnosing hospital is the large one $\left({\hat{O}}_{1}=1\right)$ for age < 55 years and the small one $\left({\hat{O}}_{1}=0\right)$ for age ≥ 55 years, implying that patients of age < 55 years benefited from large diagnosing hospitals. This reflects the delay in diagnosing stomach cancer among young patients at small hospitals, where cancer in young patients is very rare (a phenomenon called doctor's delay).

Third, we analyse the optimal regime in the population. Taking the average of $SCE(x_{11},x_{13};\;{\hat{O}}_{1},{\hat{O}}_{2})$ over $\left(x_{11},x_{13}\right)$ , we obtain the optimal SCE in the population, $\;\Gamma_{1}$ , which is estimated at 0.028 with *P*-value  =  0.237. This reveals the potential improvement for one-year survival by the optima regime.

Here, all effects are measured by the difference in mean survival, but they can also be measured by the difference in a function of mean survival. We may apply the new G-formula to estimate the causal effect measured as odds ratio, rate ratio and hazard ratio. Though the estimates are consistent, the problem of non-collapsibility of a non-linear measure becomes far worse in the context of sequential treatments, leading to biased estimates of these measures and false coverage probabilities of the confidence intervals for finite samples. Therefore, it is recommended that one uses the linear measure for the SCE.

In the medical example of this article, there was no censoring. Suppose there were non-informative censorings such as the loss of follow-up. Based on the new G-formula, we may address the loss of follow-up in the framework of single-point causal inference.^9,10^ When estimating and testing the point effects and blip effects in Sections 3.1 and 3.2, we may simply remove the censored patients in the estimation. When estimating SCEs under various regimes in Section 3.3, we may simply remove the censored patients if the loss of follow-up occurred between $Z_{1}$ and $Z_{2}$ ; if the loss of follow-up occurs after $Z_{2}$ , we may still use the data of these patients when estimating the covariate probability $P(x_{2}|x_{11},x_{13},z_{1})$ in (7).

## 4 Estimating and testing SCEs of cancer diagnosis and treatment on three-year survival

In Section 3, we replace one-year survival with three-year survival and follow the same procedure to estimate and test the blip effect and the SCE. The result is presented in Table 3.

**Table 3.** Point effects, blip effects and optimal SCEs of diagnosing and treating hospitals on three-year survival of stomach cancer: estimate, *P*-value and 95% CI.

	Estimate, *P*-value and 95% CI in column for various causal effects				
	$\vartheta_{1}$	$\vartheta_{21}$	$\vartheta_{22}$	$\vartheta_{23}$	$\vartheta_{24}$
Point	0.024	0.011	− 0.036	0.069	0.000
effect	0.413	0.882	0.728	0.168	0.997
	(−0.034,0.082)	(−0.132,0.153)	(−0.242,0.169)	(−0.029,0.168)	(−0.029,0.029)
	$\gamma_{1}$	$\gamma_{21}$	$\gamma_{22}$	$\gamma_{23}$	$\gamma_{24}$
Blip	0.014	0.011	− 0.036	0.069	0.000
effect	0.527	0.882	0.728	0.168	0.997
	(−0.029,0.058)	(−0.132,0.153)	(−0.242,0.169)	(−0.029,0.168)	(−0.029,0.029)
	$\Gamma_{1}$	$\Gamma_{21}$	$\gamma_{22}$	$\Gamma_{23}$	$\gamma_{24}$
Optimal	0.031	0.011	− 0.036	0.069	0.000
SCE	0.270	0.882	0.728	0.168	0.997
	(−0.024,0.086)	(−0.132,0.153)	(−0.242,0.169)	(−0.029,0.168)	(−0.029,0.029)

• Five point effects: $\vartheta_{1}$ of large versus small diagnosing hospital; $\vartheta_{2j}$ of large versus small treating hospital for cancer stage j=1,2,3,4.

• Five blip effects: $\gamma_{1}$ of large versus small diagnosing hospital; $\gamma_{2j}$ of large versus small treating hospital for cancer stage j=1,2,3,4.

• Five optimal SCEs: $\Gamma_{1}$ under optimal regime of diagnosing and treating hospitals; $\Gamma_{2j}$ of optimal treating hospital for cancer stage $j=1,\;3.\;\Gamma_{24}=0$ of optimal treating hospital for cancer stage 4 is the baseline, so the blip effect $\;\gamma_{24}$ is instead presented. Similarly, $\Gamma_{22}=0$ and $\gamma_{22}$ are presented.

Only for the cancer stage $x_{2}=3,$ does the large treating hospital performs better than the small one with a considerable significance: the blip effect $\gamma_{23}$ of treating hospital $z_{2}=1$ is estimated at 0.069 with *P*-value  =  0.168. For the cancer stage $x_{2}=2,$ the blip effect $\gamma_{22}$ of treating hospital $z_{2}=1$ is estimated at −0.036 with *P*-value  =  0.728; due to the low significance and the medical knowledge, we believe that the large treating hospital performs better than the small one for $x_{2}=2$ . Consequently, the estimated optimal treating hospital is the large one ${\hat{O}}_{2}=1$ for all cancer stages $x_{2}=1,\;2,\;3,\;4$ . The estimated optimal diagnosing hospital is the large one ${\hat{O}}_{1}=1$ . The SCE under the estimated optimal regime $\left({\hat{O}}_{1},{\hat{O}}_{2}\right)$ , $\Gamma_{1}$ , is estimated at 0.031 with *P*-value  =  0.270.

## 5 Comparison of our method with available methods

Here, we only consider one-year survival. Method (i) is our method described in Section 3. Method (ii) is the parametric method based on Robins’ G-formula.^2–4^ Method (iii) is the marginal structural model based on the inverse probability of treatment weighting.^4–6^ Method (iv) is the G-estimation based on SNMM or optimal-regime SNMM.^2,4,7,8^; this method incorporates both the Q-learning and A-learning in estimating optimal dynamic treatment regimes.

With our data, we aim to examine the modelling assumptions behind these methods and their abilities of estimating SCEs and comparing the underlying regimes of diagnosing and treating hospitals. These methods are active areas in the literature,^4,8^ but to focus on the problems, we do not use advanced versions of these methods, which alleviate the problems. Methods (ii)–(iv) are also described in the context of this article in Supplemental Data. See also a simulation study for a comparison between these methods.^10^

Because methods (i)–(iv) may lead to the same inference of the causal effect of treating a hospital, we focus on the causal effect of diagnosing a hospital. In Section 3, we have applied method (i) to estimate the blip effect $\gamma_{1}$ , its modification $\gamma_{1,2}$ by age and the optimal SCE, $\Gamma_{1}$ , on the population. Here, we additionally estimate the optimal blip effect of a large diagnosing hospital: $\Upsilon_{1}=SCE(D_{1}=1,{\hat{O}}_{2})-SCE(0,{\hat{O}}_{2})$ , because method (iv) does not estimate $\Gamma_{1}$ . The result from these methods is presented in Table 4. From this table, we have the following observations.

**Table 4.** Comparison of our method with available methods in Section 5: estimate, *P*-value and 95% confidence interval (95% CI) for causal effects of diagnosing and treating hospitals on one-year cancer survival.

	Estimate, *P*-value and 95% CI in column of the causal effect			
	$\gamma_{1}$	$\gamma_{1,2}$	$\Upsilon_{1}$	$\Gamma_{1}$
Method (i)	−0.009	− 0.001	− 0.010	0.028
	0.710	0.298	0.580	0.237
	(−0.054,0.037)	(−0.003,0.001)	(−0.059,0.038)	(−0.019,0.075)
Method (ii)	−0.340		− 0.155	− 0.012
	0.0		0.005	0.798
	(−0.416,−0.265)		(−0.263,−0.048)	(−0.102,−0.079)
Method (iii)	0.290		− 0.030	
	0.299		0.864	
	(−0.257,0.836)		(−0.375,0.315)	
Method (iv)	−0.009	− 0.003	− 0.011	
	0.713	0.322	0.662	
	(−0.054,0.037)	(−0.009,0.003)	(−0.059,0.038)	

• Four estimation methods: method (i) our method; method (ii) the parametric method based on Robins’ G-formula; (iii) the marginal structural model based on inverse probability of treatment weighting; method (iv) the G-estimation based on SNMM or optimal-regime SNMM; Empty cells imply that they are not easily estimable by the method.

• Causal effects: $\gamma_{1}$ the blip effect of diagnosing hospital; $\gamma_{1,2}$ the modification of $\gamma_{1}$ by age; $\Upsilon_{1}$ the optimal blip effect of diagnosing hospital; $\Gamma_{1}$ the SCE under the optimal regime of diagnosing and treating hospitals described in Section 5.

With method (i), the modelling assumptions for the outcome are models (1) and (2) for estimating the point effects of $z_{1}=1$ and $z_{2}=1$ and SNMM (3) for estimating and testing the blip effects. When estimating and testing $\gamma_{1},$ $\gamma_{1,2},$ $\Gamma_{1}$ and $\Upsilon_{1},$ a total of six parameters: $\vartheta_{1}$ for the point effect of $z_{1}=1$ and $\left(\vartheta_{21,1},\vartheta_{21,2},\;\vartheta_{22},\;\vartheta_{23},\;\vartheta_{24}\right)$ for the point effect of $z_{2}=1$ , are involved. Method (i) yields the estimates for $\gamma_{1}=-0.009$ , $\gamma_{1,2}=-0.001$ , $\Upsilon_{1}=-0.010$ and $\Gamma_{1}=0.028$ in line of the medical knowledge. It also yields the estimate for the optimal diagnosing hospital: a large one $\left({\hat{O}}_{1}=1\right)$ for age < 55 years and a small one $\left({\hat{O}}_{1}=0\right)$ for age ≥ 55 years, which indicates the delay in diagnosing young patients at small hospitals (the phenomenon of doctor's delay). Method (i) allows for comparison between regimes $\left(O_{1},O_{2}\right)$ and (0,0) by testing $\Gamma_{1}$ . In general, it allows for comparison between different regimes, because their SCEs are estimated under the same modelling assumption.

With method (ii), we assume an unsaturated outcome model for the standard parameters $\mu (x_{11},x_{13},z_{1},x_{2},z_{2})$ as a trade-off between efficiency and bias, where $x_{13}$ is categorized as lower than the median or higher. Even for this unsaturated model, a total of 2×2×2×4×2=64 standard parameters are involved in estimating and testing $\gamma_{1},$ $\gamma_{1,2},$ $\Gamma_{1}$ and $\Upsilon_{1}$ . Method (ii) yields the estimates ${\hat{\gamma }}_{1}=-0.340$ , ${\hat{\Upsilon }}_{1}=-0.155$ and ${\hat{\Gamma }}_{1}=-0.012$ . The estimated optimal diagnosing hospital is the small one $\left({\hat{O}}_{1}=0\right)$ for all ages. Method (ii) allows for comparison between regimes $\left(O_{1},O_{2}\right)$ and (0,0) by testing $\Gamma_{1}$ , because the means of the potential outcomes under the two regimes are estimated under the same model for $\mu (x_{11},x_{13},z_{1},x_{2},z_{2}).$ However, this method is not able to estimate $\gamma_{1,2}$ due to the use of the unsaturated model. Furthermore, according to the medical knowledge, the estimates ${\hat{\gamma }}_{1}$ and ${\hat{\Upsilon }}_{1}$ cannot possibly be true as the differences in one-year survival between large and small diagnosing hospitals. The estimate ${\hat{\Gamma }}_{1}$ is also biased, because the optimal SCE, $\Gamma_{1},\;$ should be larger than or equal to zero.

With method (iii), we assume models for the probabilities of assigning diagnosing and treating hospitals and use the estimated probabilities to calculate stabilized weight and non-stabilized weight for the outcome. With the stabilized weighted outcome, we estimate the mean of the potential outcome under a static regime and thus $\gamma_{1}$ . With the non-stabilized weighted outcome, we estimate the mean of the potential outcome under a dynamic regime and thus $\Upsilon_{1}$ . Method (iii) yields the estimate ${\hat{\gamma }}_{1}=0.290$ . The estimate is not medically sensible possibly due to an imbalance between $\left(z_{1},z_{2}\right)$ and the covariates in the data.^4–6^ Method (iii) also yields ${\hat{\Upsilon }}_{1}=-0.030.$ The estimated optimal diagnosing hospital is the small one $\left({\hat{O}}_{1}=0\right)$ for all ages. Method (iii) is not able to estimate $\gamma_{1,2}$ due to an imbalance between $\left(z_{1},z_{2}\right)$ and the covariates in the data.^4,6^ Method (iii) does not estimate $\Gamma_{1}$ either, which compares between the dynamic regime $\left(O_{1},O_{2}\right)$ and static regime (0,0).

With method (iv), to estimate $\gamma_{1}$ and $\gamma_{1,2}$ , we assume SNMM (3) and a model for the mean of the potential outcome under regime (0,0), namely, $E\{\mu (x_{11},x_{13},z_{1}=0,x_{2},z_{2}=0)|x_{11},x_{13},z_{1}=0\}$ with respect to $P(x_{2}|x_{11},x_{13},z_{1}=0)$ according to Robins’ G-formula.^1,2^ To estimate $\Upsilon_{1},$ we assume an optimal-regime SNMM and a model for the mean of the potential outcome under regime $(0,\;{\hat{O}}_{2}),$ namely, $E\{\mu (x_{11},x_{13},z_{1}=0,x_{2},z_{2})|x_{11},x_{13},z_{1}=0\}$ with respect to $P(x_{2}|x_{11},x_{13},z_{1}=0)$ according to Robins’ G-formula, where ${\hat{O}}_{2}$ deterministically and potentially assigned $z_{2}$ .^1,2^ Method (iv) yields nearly the same estimates for $\gamma_{1}$ and $\gamma_{1,2}$ , and $\Upsilon_{1}$ as method (i), albeit with lower significance. The estimated optimal diagnosing hospital is the large one $\left({\hat{O}}_{1}=1\right)$ for age < 68 years and the small one $\left({\hat{O}}_{1}=0\right)$ for age ≥ 68 years; it is different from the one obtained from method (i), possibly due to the misspecification of the model for the baseline. Method (iv) does not estimate $\Gamma_{1}$ , so it is difficult to compare between regimes $\left(O_{1},O_{2}\right)$ and (0,0). Generally, it is difficult to compare between different regimes, because their SCEs are estimated under different SNMMs. It is also difficult to specify models for the baselines.

## 6 Conclusion

In recent years, a huge amount of clinical data has become available, for instance, from various Swedish quality registers, which contain almost all economic and social information of a patient as well as a nearly complete record of clinical visits for the diagnoses and treatments of various cancers. Such data should lead to a large variety of comprehensive longitudinal studies of the influences of early diagnosis on various cancer outcomes such as survival and progression. In these studies, one needs not only to estimate SCEs under various regimes of diagnoses and treatments but also to compare these regimes.

In this article, we study the application of a parametric likelihood-based approach,^10^ which allows for not only an unbiased estimation of SCEs under various regimes but also a comparison between these regimes by testing these SCEs under the same modelling assumption. Our method is implemented in three steps: first to estimate and test the point effect, second to use the estimated point effect to estimate and test the blip effect, and finally use the estimated blip effects to estimate and test SCEs. Each of these steps can be carried out using the usual regression and can be examined using the usual modelling tools, familiar to epidemiologists, in the causal inference of single-point treatment. Please note that the SCE is such a parameter that involves the entire data-generating mechanism of all covariates, treatments and the outcome across different diagnosing and treating stages, and consequently, it is highly difficult for a single procedure or algorithm to achieve both reliable estimation and hypothesis testing of the SCE.

Our medical example contains most of the essential components for evaluating the performance of cancer diagnosis: the blip effect, its modification by covariates and SCE under a general regime. It is also an observational study with continuous covariates. With its simple setting, we can readily extend this example to various cancer types; to different cancer outcomes such as cancer progression, quality of life and others; to different diagnosing techniques such as the biomarkers or a sequence of screening steps; to different modification factors such as social-economic status, comorbidity, and family history. With complex covariate settings in cancer diagnoses, we believe that we may also apply more advanced methods than the usual regression, such as the targeted maximum likelihood method and even machine learning, to estimate the point effects.^11^ If it is difficult to specify the distribution of the observable variables, we believe that we may also employ semi-parametric or non-parametric methods to estimate the point effects.

## Supplemental Material

sj-docx-1-smm-10.1177_09622802221098429 - Supplemental material for Estimating and testing the influence of early diagnosis on cancer survival via point effects of diagnoses and treatments Click here for additional data file. Supplemental material, sj-docx-1-smm-10.1177_09622802221098429 for Estimating and testing the influence of early diagnosis on cancer survival via point effects of diagnoses and treatments by Xiaoqin Wang, Johannes Blom, Weimin Ye and Li Yin in Statistical Methods in Medical Research
