# Supplementary material for: Estimating and testing the influence of early diagnosis on cancer survival via point effects of diagnoses and treatments
Source: Stat Methods Med Res. 2022 May 4;31(8):1538–48. doi: 10.1177/09622802221098429 (PMC9315175; doi:10.1177/09622802221098429)
Supplement: sj-docx-1-smm-10.1177_09622802221098429 - Supplemental material for Estimating and testing the influence of early diagnosis on cancer survival via point effects of diagnoses and treatments [file sj-docx-1-smm-10.1177_09622802221098429.docx]

**Supplemental Data to “Estimating and testing influence of early diagnosis on cancer survival via point effects of diagnoses and treatments”**

Xiaoqin Wang, Johannes Blom, Weimin Ye, Li Yin

1. **General description of the new G-formula for estimating and testing SCEs via point effects of treatments**
   1. **Potential and observable variables**

Let $D_{t}$ at time $t=1, \ldots, T$ be the treatment variable, which potentially and deterministically assigned treatments $z_{t}$ to each unit of the population. A treatment regime is a sequence of such treatment variables, $\boldsymbol{D}_{1}^{T}=(D_{1},\ldots, D_{T})$. Prior to $D_{1}$, there exists a set $\boldsymbol{X}_{1}$ of stationary covariates. Under $\boldsymbol{D}_{1}^{T}$, each unit could have a set $\boldsymbol{X}_{t}(\boldsymbol{D}_{1}^{t-1})$ of potential time-dependent covariates between $D_{t-1}$ and $D_{t}$ ($t=2, \ldots, T$) and a potential outcome $Y(\boldsymbol{D}_{1}^{T})$ of interest after the last treatment $D_{T}$.

All potential variables in the temporal order are $\{\boldsymbol{X}_{1},D_{1},\boldsymbol{X}_{2}\left( D_{1} \right),D_{2},\ldots,\boldsymbol{X}_{T}\left( \boldsymbol{D}_{1}^{T-1} \right),D_{T},Y(\boldsymbol{D}_{1}^{T})\}$ while their realizations are ${\boldsymbol{\{x}}_{1},z_{1},\boldsymbol{x}_{2},z_{2},\ldots,\boldsymbol{x}_{T},z_{T},y\}$. For notational convenience, let $\boldsymbol{D}_{1}^{t}=(D_{1},\ldots, D_{t})$and $\boldsymbol{X}_{1}^{t}\left( \boldsymbol{D}_{1}^{t-1} \right)=\{\boldsymbol{X}_{1},\boldsymbol{X}_{2}\left( D_{1} \right),\ldots, \boldsymbol{X}_{t}(\boldsymbol{D}_{1}^{t-1})\}$ as well as $\boldsymbol{z}_{1}^{t}=(z_{1},\ldots, z_{t})$and $\boldsymbol{x}_{1}^{t}=\{\boldsymbol{x}_{1},\boldsymbol{x}_{2},\ldots, \boldsymbol{x}_{t}\}$. Given $(\boldsymbol{x}_{1}^{t},\boldsymbol{z}_{1}^{t-1})$, we document the treatment regime as $\boldsymbol{D}_{t}^{T}=(D_{t},\ldots, D_{T})$, its potential covariates as $\boldsymbol{X}_{t+1}^{T}\left( \boldsymbol{D}_{t}^{T-1} \right)$ and potential outcome as $Y(\boldsymbol{D}_{t}^{T})$.

Now we consider observable variables. Let $Z_{t}$ be the treatment variable at $t=1, \ldots, T$; $\boldsymbol{X}_{1}$be the set of stationary covariates; $\boldsymbol{X}_{t}$ be the set of time-dependent covariates between $Z_{t-1}$ and $Z_{t}$ ($t=2, \ldots, T$); and $Y$ be the outcome of interest. All observable variables in the temporal order are $(\boldsymbol{X}_{1},Z_{1},\boldsymbol{X}_{2},Z_{2},\ldots, \boldsymbol{X}_{T},Z_{T},Y)$ while their observed values are $(\boldsymbol{x}_{1},z_{1},\boldsymbol{x}_{2},z_{2},\ldots, \boldsymbol{x}_{T},z_{T},y)$. For notational convenience, let $\boldsymbol{Z}_{1}^{t}=(Z_{1},\ldots, Z_{t})$ and $\boldsymbol{X}_{1}^{t}=(\boldsymbol{X}_{1},\ldots,\boldsymbol{X}_{t})$ as well as $\boldsymbol{z}_{1}^{t}=(z_{1},\ldots, z_{t})$ and $\boldsymbol{x}_{1}^{t}=(\boldsymbol{x}_{1},\ldots, \boldsymbol{x}_{t})$. In the following, we will use $P(.)$ to denote the probability distribution of a discrete variable or the density distribution of a continuous variable. The joint distribution of $(\boldsymbol{X}_{1}^{T}, \boldsymbol{Z}_{1}^{T}, Y)$ is given by

$$P\left( \boldsymbol{x}_{1}^{T},\boldsymbol{z}_{1}^{T},y \right)=P(\boldsymbol{x}_{1})P(z_{1}|\boldsymbol{x}_{1})\cdots P\left( \boldsymbol{x}_{T} \right|\boldsymbol{x}_{1}^{T-1},\boldsymbol{z}_{1}^{T-1})P(z_{T}\left| \boldsymbol{x}_{1}^{T},\boldsymbol{z}_{1}^{T-1} \right)P\left( y | \boldsymbol{x}_{1}^{T},\boldsymbol{z}_{1}^{T} \right)$$

The standard parameters for these conditional distributions are simply their conditional expectations, for instance, the standard parameter for $P(y|\boldsymbol{x}_{1}^{T},\boldsymbol{z}_{1}^{T})$ is the conditional mean $E(Y|\boldsymbol{x}_{1}^{T},\boldsymbol{z}_{1}^{T})$, denoted by $\mu(\boldsymbol{x}_{1}^{T},\boldsymbol{z}_{1}^{T})$.

To identify the potential variables {$\boldsymbol{X}_{1}^{T}\left( \boldsymbol{D}_{1}^{T-1} \right), \boldsymbol{D}_{1}^{T}, Y(\boldsymbol{D}_{1}^{T})\}$ by the observable variables $(\boldsymbol{X}_{1}^{T}, \boldsymbol{Z}_{1}^{T}, Y)$, Robins introduced the identifying condition which consists of the consistency assumption, the positivity assumption for treatments and the assumption of no unmeasured confounders.^1,2^ The identifying condition is satisfied in sequential randomized experiments where treatment $z_{t}$ is randomly assigned according to a history $\boldsymbol{(x}_{1}^{\boldsymbol{t}-1}, \boldsymbol{z}_{\boldsymbol{1}}^{t-1}$**)** of the earlier covariates and treatments. It is approximately satisfied in observational studies with a sufficient number of covariates. Throughout, we assume the identifying condition.

- 1. **New G-formula to estimate and test the blip effect**

In causal inference, we aim at the causal effect under a specified regime of treatments, shorthanded as sequential causal effect (SCE). Here, we consider a special type of SCEs, called the blip effects, from which all other SCEs are determined.^9,10^ Under the identifying condition, Wang and Yin derived the new G-formula for estimating and testing the blip effect, as summarized below.^9,10^

Let $\mu\left( \boldsymbol{x}_{1}^{t}, \boldsymbol{z}_{\boldsymbol{1}}^{t-1}, z_{t} \right)=E(Y|\boldsymbol{x}_{1}^{t}, \boldsymbol{z}_{\boldsymbol{1}}^{t-1}, z_{t})$ be the conditional mean of observable outcome $Y$ given $(\boldsymbol{x}_{1}^{t}, \boldsymbol{z}_{\boldsymbol{1}}^{t-1}, z_{t})$. Without loss of generality, we take $z_{t}=0$ as control treatment and $\boldsymbol{z}_{\boldsymbol{t}}^{T}\boldsymbol{=0=}\left( z_{t}=0,\ldots,z_{T}=0 \right)$ as control regime. Then the point effect of treatment $z_{t}$ in stratum $(\boldsymbol{x}_{1}^{t}, \boldsymbol{z}_{\boldsymbol{1}}^{t-1})$ is the following difference between the means

$$\vartheta\left( \boldsymbol{x}_{1}^{t}, \boldsymbol{z}_{\boldsymbol{1}}^{t-1};z_{t} \right)=\mu\left( \boldsymbol{x}_{1}^{t}, \boldsymbol{z}_{\boldsymbol{1}}^{t-1}, z_{t} \right)-\mu\left( \boldsymbol{x}_{1}^{t}, \boldsymbol{z}_{\boldsymbol{1}}^{t-1}, 0 \right) (8)$$

Clearly, $\vartheta\left( \boldsymbol{x}_{1}^{t}, \boldsymbol{z}_{\boldsymbol{1}}^{t-1}; 0 \right)=0.$ The point effect is simply the point effect of treatment $z_{t}$ in causal inference of single-point treatment and can be estimated by the usual regression.

The blip effect $\phi\left( \boldsymbol{x}_{1}^{t}, \boldsymbol{z}_{\boldsymbol{1}}^{t-1}; z_{t} \right)$ of treatment $z_{t}$ in the stratum $(\boldsymbol{x}_{1}^{t}, \boldsymbol{z}_{\boldsymbol{1}}^{t-1})$ is an increase of the mean of potential outcome $Y(\boldsymbol{D}_{t}^{T})$ when potentially applying regimes $\boldsymbol{D}_{t}^{T}=(z_{t},\boldsymbol{0})$ versus $(0,\boldsymbol{0)}$ to the stratum. That is,

$$\phi\left( \boldsymbol{x}_{1}^{t}, \boldsymbol{z}_{\boldsymbol{1}}^{t-1}; z_{t} \right)=E\left\{ Y\left( z_{t},\boldsymbol{0} \right)|\boldsymbol{x}_{1}^{t}, \boldsymbol{z}_{\boldsymbol{1}}^{t-1} \right\}-E\left\{ Y\left( 0\boldsymbol{,0} \right)|\boldsymbol{x}_{1}^{t}, \boldsymbol{z}_{\boldsymbol{1}}^{t-1} \right\}.$$

The blip effect is also called the net effect.^9^ Clearly, we have $\phi\left( \boldsymbol{x}_{1}^{t}, \boldsymbol{z}_{\boldsymbol{1}}^{t-1}; 0 \right)=0.$ Oftentimes, the blip effects follow a certain pattern described by structural nested mean model (SNMM),

$$\phi\left( \boldsymbol{x}_{1}^{t}, \boldsymbol{z}_{\boldsymbol{1}}^{t-1}; z_{t} \right)=f\left( \boldsymbol{x}_{1}^{t}, \boldsymbol{z}_{\boldsymbol{1}}^{t-1}, z_{t}; \boldsymbol{\gamma} \right), t=1,\ldots,T (9)$$

where $f(.)$ is a deterministic function of $\boldsymbol{(x}_{1}^{t}, \boldsymbol{z}_{\boldsymbol{1}}^{t-1}, z_{t})$ and is indexed by a parameter vector $\boldsymbol{\gamma}$ of small dimension.^2,4^

Intuitively, the point effect $\vartheta\left( \boldsymbol{x}_{1}^{t}, \boldsymbol{z}_{\boldsymbol{1}}^{t-1}; z_{t} \right)$ is the result of treatment $z_{t}$ and the subsequent treatments $\boldsymbol{Z}_{\boldsymbol{t+1}}^{\boldsymbol{T}}$**,** and it may decompose into a sum of the blip effects of these treatments**.** Therefore, we should have the following new G-formula for the blip effect

$$\vartheta\left( \boldsymbol{x}_{1}^{t}, \boldsymbol{z}_{\boldsymbol{1}}^{t-1}; z_{t} \right)=\phi\left( \boldsymbol{x}_{1}^{t}, \boldsymbol{z}_{\boldsymbol{1}}^{t-1};z_{t} \right)+ (10)$$

$$\sum_{s=t+1}^{T} E\{\phi\left( \boldsymbol{x}_{1}^{s}, \boldsymbol{z}_{\boldsymbol{1}}^{s-1};z_{s} \right)|\boldsymbol{x}_{1}^{t}, \boldsymbol{z}_{\boldsymbol{1}}^{t-1}, z_{t}\}-\sum_{s=t+1}^{T} E\{\phi\left( \boldsymbol{x}_{1}^{s}, \boldsymbol{z}_{\boldsymbol{1}}^{s-1}; z_{s} \right)|\boldsymbol{x}_{1}^{t}, \boldsymbol{z}_{\boldsymbol{1}}^{t-1}, 0\}$$

where the expectations are with respect to $P(\boldsymbol{x}_{t+1}^{s}, \boldsymbol{z}_{\boldsymbol{t+1}}^{s-1}, z_{s}|\boldsymbol{x}_{1}^{t}, \boldsymbol{z}_{\boldsymbol{1}}^{t-1}, z_{t})$ and $P(\boldsymbol{x}_{t+1}^{s}, \boldsymbol{z}_{\boldsymbol{t+1}}^{s-1}, z_{s}|\boldsymbol{x}_{1}^{t}, \boldsymbol{z}_{\boldsymbol{1}}^{t-1}, 0)$. In particular, at last time $t=T$, we have

$$\vartheta\left( \boldsymbol{x}_{1}^{T}, \boldsymbol{z}_{\boldsymbol{1}}^{T-1}; z_{T} \right)=\phi\left( \boldsymbol{x}_{1}^{T}, \boldsymbol{z}_{\boldsymbol{1}}^{T-1};z_{T} \right)$$

Theoretically, Wang and Yin proved the above new G-formula (10).^9,10^ Formula (10) implies that point effect of $z_{t}$ consists of the blip effects of $z_{t}$ and $\boldsymbol{z}_{\boldsymbol{t+1}}^{T}$ and can be used to obtain the blip effects. In comparison, Robins’ G-formula expresses the blip effects in terms of the standard parameters $\mu(\boldsymbol{x}_{1}^{T},\boldsymbol{z}_{1}^{T})$^1,2^ .

To estimate and test $\boldsymbol{\gamma}$ in SNMM (9), we insert (9) into (10) and treat the obtained equation as a regression model, where the response variables are the estimated point effects and the explanatory variables are the observed proportions corresponding to the probabilities. In the regression, we need the conditional variance of the estimated point effect given all covariates and treatments $(\boldsymbol{x}_{1}^{T}, \boldsymbol{z}_{\boldsymbol{1}}^{T}\boldsymbol{)}$**,** which is obtained by specifying a constant dispersion parameter for the distribution $P\left( y | \boldsymbol{x}_{1}^{T},\boldsymbol{z}_{1}^{T} \right)$ . Noticeably, the conditional covariance between the estimated point effects at different times is negligible, for instance, it is equal to zero for the normal outcome.^9,10^ The bootstrap method is used to obtain the covariance matrix $\text{cov}\text{(}\hat{\boldsymbol{\gamma}}\text{)}$ incorporating the variability of $(\boldsymbol{x}_{1}^{T}, \boldsymbol{z}_{\boldsymbol{1}}^{T}\boldsymbol{)}$. With $\hat{\boldsymbol{\gamma}}$ and $\text{cov}\text{(}\hat{\boldsymbol{\gamma}}\text{)}$, we conduct the Wald test on $\boldsymbol{\gamma}$.

- 1. **New G-formula to estimate and test SCEs**

The SCE under regime $\boldsymbol{D}_{t}^{T}$ in stratum $(\boldsymbol{x}_{1}^{t}, \boldsymbol{z}_{\boldsymbol{1}}^{t-1}\boldsymbol{)}$, $\text{SCE}\left( \boldsymbol{x}_{1}^{t}, \boldsymbol{z}_{1}^{t-1}; \boldsymbol{D}_{t}^{T} \right)$, is an increase of the mean of potential outcome $Y\left( \boldsymbol{D}_{t}^{T} \right)$ when potentially applying regime $\boldsymbol{D}_{t}^{T}$ versus $\boldsymbol{0}$ to the stratum, namely,

$$\text{SCE(}\boldsymbol{x}_{1}^{t}, \boldsymbol{z}_{\boldsymbol{1}}^{t-1}; \boldsymbol{D}_{t}^{T} )=E\left\{ Y\left( \boldsymbol{D}_{t}^{\boldsymbol{T}} \right)\boldsymbol{|}\boldsymbol{x}_{1}^{t}, \boldsymbol{z}_{\boldsymbol{1}}^{t-1} \right\}-E\left\{ Y\left( \boldsymbol{0} \right)\boldsymbol{|}\boldsymbol{x}_{1}^{t}, \boldsymbol{z}_{\boldsymbol{1}}^{t-1} \right\}.$$

By applying formula (18) in Theorem 2 of Wang and Yin^10^ to $\boldsymbol{A}_{t}^{T}= \boldsymbol{D}_{t}^{T}$ and $\boldsymbol{B}_{t}^{T}= \boldsymbol{0}$ and noticing that $\phi\left( \boldsymbol{x}_{1}^{s}, \boldsymbol{z}_{\boldsymbol{1}}^{s-1}; 0 \right)=0,$we obtain the following new G-formula for the SCE

$$\text{SCE}\left( \boldsymbol{x}_{1}^{t}, \boldsymbol{z}_{\boldsymbol{1}}^{t-1}; \boldsymbol{D}_{t}^{T} \right)=\phi\left( \boldsymbol{x}_{1}^{t}, \boldsymbol{z}_{\boldsymbol{1}}^{t-1}; z_{t} \right)+\sum_{s=t+1}^{T} E\{\phi\left( \boldsymbol{x}_{1}^{s}, \boldsymbol{z}_{\boldsymbol{1}}^{s-1}; z_{s} \right)|\boldsymbol{x}_{1}^{t}, \boldsymbol{z}_{\boldsymbol{1}}^{t-1}, z_{t}\}, (11)$$

where the expectation is with respect to the distribution of observable covariates $\boldsymbol{x}_{t+1}^{s}:$ $\prod_{k=t+1}^{s} P\left( \boldsymbol{x}_{k} \right|\boldsymbol{x}_{1}^{k-1},\boldsymbol{z}_{1}^{k-1})$. In particular, at the last time $t=T$, we have

$$\text{SCE}\left( \boldsymbol{x}_{1}^{T}, \boldsymbol{z}_{\boldsymbol{1}}^{T-1}; D_{T} \right)=\phi\left( \boldsymbol{x}_{1}^{T}, \boldsymbol{z}_{\boldsymbol{1}}^{T-1}; z_{T} \right).$$

The formula (11) implies that all SCEs are determined by blip effects. In comparison, Robins’ G-formula expresses the SCE in terms of the standard parameters $\mu(\boldsymbol{x}_{1}^{T},\boldsymbol{z}_{1}^{T})$.^1,2^

To estimate and test SCEs, we need to estimate the covariate probability $P\left( \boldsymbol{x}_{k} \right|\boldsymbol{x}_{1}^{k-1},\boldsymbol{z}_{1}^{k-1})$ by the usual regression in the framework of single-point causal inference. Because covariate $\boldsymbol{x}_{k}$ is of secondary importance, there is a great flexibility in estimating the covariate probability. For instance, the covariate probability often depends on a short history of treatments and covariates, say, $(\boldsymbol{x}_{k-1}, z_{k-1})$, so that we only need to estimate $P\left( \boldsymbol{x}_{k} \right|\boldsymbol{x}_{k-1}, z_{k-1})$. With the estimates of the covariate probabilities and the estimates of the blip effects obtained in the previous subsection, we may estimate and test SCEs under various regimes including the optimal ones. Please notice that we estimate and test all blip effects and SCEs under one common SNMM, namely SNMM (9).

1. **Description of available methods (ii), (iii) and (iv) in Section 5.**

As described in Section 3.1 of the paper, we obtain model (2) for the mean $\mu\left( x_{11},x_{13},x_{2},z_{2} \right)$, based on which we obtain the estimate $\hat{\vartheta}\left( x_{11},x_{13},x_{2}; z_{2} \right)$ for the point effect of treating hospital $z_{2}$. Because the treating hospital $Z_{2}$ is the last treatment variable, $\hat{\vartheta}\left( x_{11},x_{13},x_{2}; z_{2} \right)$ is equal to the estimate $\hat{\phi}\left( x_{11},x_{13},z_{1},x_{2};z_{2} \right)$ for the blip effect of $z_{2}$, which is equal to the estimate $\hat{SCE}\left( x_{11},x_{13},z_{1},x_{2};D_{2} \right)$ for the SCE under regime $D_{2}$ assigning $z_{2}.$ By using $\hat{\mu}\left( x_{11},x_{13},x_{2},z_{2} \right),$ we obtain the estimate $\hat{O}_{2}=\text{arg ma}x_{z_{2}}\hat{\mu}\left( x_{11},x_{13},x_{2},z_{2} \right)$ for the optimal treating hospital, which is the same as $\hat{O}_{2}=\text{arg ma}x_{D_{2}}\hat{SCE}\left( x_{11},x_{13},z_{1},x_{2};D_{2} \right)$ obtained by using $\hat{SCE}\left( x_{11},x_{13},z_{1},x_{2};D_{2} \right)$in Section 3.3.

Here, we focus on the causal effect of diagnosing hospital $z_{1}=1$. We assume the same identifying condition for methods (ii)-(iv) as it is for method (i) (our method). As a result, we can use methods (ii)-(iv) to identify and estimate the blip effect and the SCE by observed data.

- 1. **Method (ii): parametric method based on Robin’s G-formula.**

The parametric method is constructed using Robins’ G-formula.^2,3^ By the usual regression, we found that gender $x_{11}$, age $x_{13}$ and cancer stage $x_{2}$ are significant to the survival $Y$ and so obtained the standard parameter $\mu\left( x_{11},x_{13},{z_{1},x}_{2},z_{2} \right)=E(Y|x_{11},x_{13},{z_{1},x}_{2},z_{2})$. Under the identifying condition, Robins’ G-formula expresses the mean of the potential outcome $Y(D_{1},D_{2})$ in stratum ($x_{11},x_{13})$ in terms of the standard parameters by

$$E\left\{ Y\left( D_{1},D_{2} \right)|x_{11},x_{13} \right\}=\sum_{x_{2}} \mu\left( x_{11},x_{13},{z_{1},x}_{2},z_{2} \right)P\left( x_{2} | x_{11},x_{13},z_{1} \right) (12)$$

where regime $(D_{1},D_{2})$ potentially and deterministically assigned diagnosing and treating hospitals $(z_{1},z_{2})$ and it can noticeably be dynamic regime. Robins’ G-formula for the mean of the potential outcome $Y(D_{1},D_{2})$ in the population is

$$E\left\{ Y\left( D_{1},D_{2} \right) \right\}=\sum_{x_{11},x_{13},x_{2}} \mu(x_{11},x_{13},{z_{1},x}_{2},z_{2})P\left( x_{2} | x_{11},x_{13},z_{1} \right)P(x_{11},x_{13}). (13)$$

With method (ii), one sets up an unsaturated model for the standard parameters $\mu(x_{11},x_{13},{z_{1},x}_{2},z_{2})$ as a tradeoff between efficiency and bias. Here, we parametrize $\mu(x_{11},x_{13},{z_{1},x}_{2},z_{2})$ by categorizing $x_{13}$ into lower than the median or higher. The probability $P\left( x_{2} | x_{11},x_{13},z_{1} \right)$ is modeled in the framework of single-point causal inference by assuming a parametric model and the multinomial distribution. With the estimates for $\mu(x_{11},x_{13},{z_{1},x}_{2},z_{2})$ and $P\left( x_{2} | x_{11},x_{13},z_{1} \right)$, we estimate the following SCEs.

Applying (13) to regimes ${(D}_{1},D_{2})=(1, 0)$ and $(0, 0)$, we estimate the blip effect of $z_{1}=1$ in the population: $\gamma_{1}=E\left\{ Y\left( 1, 0 \right) \right\}-\left\{ Y\left( 0, 0 \right) \right\}$. As described at the beginning of this section, we have already obtained the estimate $\hat{O}_{2}$ of the optimal treating hospital. Applying (13) to regimes ${(D}_{1},D_{2})=(1,\hat{O}_{2})$ and $(0,\hat{O}_{2})$, we estimate the optimal blip effect of $z_{1}=1$ in the population: $\Upsilon_{1}=E\left\{ Y\left( 1, \hat{O}_{2} \right) \right\}-E\left\{ Y\left( 0,\hat{O}_{2} \right) \right\}$. From $\hat{\Upsilon}_{1}$ , we estimate the optimal diagnosing hospital $O_{1}$ as $\hat{O}_{1}=0$ (small diagnosing hospital). Applying (13) to regimes ${(D}_{1},D_{2})=(\hat{O}_{1},\hat{O}_{2})$ and $(0, 0)$, we estimate the optimal SCE: $\Gamma_{1}=\left\{ Y\left( \hat{O}_{1},\hat{O}_{2} \right) \right\}-\left\{ Y\left( 0,0 \right) \right\}$. Due to use of the unsaturated model for $\mu(x_{11},x_{13},{z_{1},x}_{2},z_{2})$, we were not able to apply (12) to estimate the modification of $\gamma_{1}$, $\Upsilon_{1}$ or $\Gamma_{1}$ by age $x_{13}.$

Due to the null paradox, the unsaturated model for the standard parameters $\mu(x_{11},x_{13},{z_{1},x}_{2},z_{2})$ leads to considerable biases for $\gamma_{1}$, $\Upsilon_{1}$ and $\Gamma_{1}$. Furthermore, the parameters $\gamma_{1}$, $\Upsilon_{1}$ and $\Gamma_{1}$ have wide confidence intervals, due to the need to estimate a total of $2\times2\times2\times4\times2=64$ standard parameters $\mu(x_{11},x_{13},{z_{1},x}_{2},z_{2})$.

In comparison, method (i) does not impose equalities between the standard parameters and so the null paradox does not occur. Furthermore, When estimating $\gamma_{1}$, $\Upsilon_{1}$ and $\Gamma_{1}$, method (i) only needs to estimate a total of six parameters: $\vartheta_{1}$ for the point effect of $z_{1}=1$ and $(\vartheta_{21,1},\vartheta_{21,2}, \vartheta_{22}, \vartheta_{23}, \vartheta_{24})$ for the point effect of $z_{2}=1$, as described in Section 3.1 of the paper. For a long sequence of cancer diagnoses and treatments, the problem becomes far worse with the null paradox and the curse of dimensionality, and in this case, method (i) may provide a useful tool for estimating and testing the causal effect of cancer diagnosis.^9,10^

**2.2 Method (iii): marginal structural model based on inverse probability of treatment weighting**

The marginal structural model is constructed by weighting each observation with the inverse probability of treatment.^4-6^

First, we estimate the blip effect of diagnosing hospital $z_{1}$. By the usual logistic model, we find that geographic area $x_{12}$, age $x_{13}$, diagnosing hospital $z_{1}$ and cancer stage $x_{2}$ are significant to the assignment of treating hospital $z_{2}$, and obtain the estimates for $P\left( z_{2} | x_{12},x_{13},z_{1} \right)$ and $P\left( z_{2} | x_{12},x_{13},z_{1},x_{2} \right)$. With these estimates, we obtain the estimate for the stabilized weight

$$sw\left( x_{12},x_{13},z_{1},x_{2},z_{2} \right)=\frac{P\left( z_{2} | x_{12},x_{13},z_{1} \right)}{P\left( z_{2} | x_{12},x_{13},z_{1},x_{2} \right)}.$$

With this weight, we obtain the weighted outcome $Y^{sw}$. Under the identifying condition, we have

$$E\left\{ Y\left( D_{1}, D_{2} \right) | x_{12},x_{13} \right\}=E\left( Y^{sw} | x_{12},x_{13},z_{1},z_{2} \right). (14)$$

where regime $\left( D_{1},D_{2} \right)$ potentially and deterministically assigned $(z_{1},z_{2})$ to stratum $(x_{12},x_{13})$. Taking average with respect to ${P(x}_{12},x_{13})$, we obtain

$$E\left\{ Y\left( D_{1},D_{2} \right) \right\}=E\left( Y^{sw} | z_{1},z_{2} \right). (15)$$

Applying (15) to regimes ${(D}_{1},D_{2})=(1, 0)$ and $(0, 0)$, we estimate the blip effect of large diagnosing hospital: $\gamma_{1}=E\left\{ Y\left( 1, 0 \right) \right\}-E\left\{ Y\left( 0, 0 \right) \right\}$. However, due to imbalanced data between $(z_{1},z_{2})$ and the covariates, we were not able to apply (14) to the two regimes to estimate the modification of $\gamma_{1}$ by age $x_{13}$; this phenomenon due to imbalanced data is well studied in the literature.^4-6^ Noticeably, the stabilized weight cannot be used to estimate SCEs under dynamic regimes, for instance, the optimal blip effect below.^4-6^

Second, we estimate the optimal blip effect of $z_{1}=1$. By the usual logistic regression, we find that only geographic area $x_{12}$ is significant to the assignment of diagnosing hospital $z_{1}$ and $(x_{12},x_{13},z_{1},x_{2})$ to treating hospital $z_{2}$. As a result, we obtain the estimates for $P\left( z_{1} | x_{12} \right)$ as well as $P\left( z_{2} | x_{12},x_{13},z_{1},x_{2} \right)$. With these estimates, we obtain the estimate for the non-stabilized weight

$$nw\left( x_{12},x_{13},z_{1},x_{2},z_{2} \right)=\frac{1}{P\left( z_{2} | x_{12},x_{13},z_{1},x_{2} \right)P\left( z_{1} | x_{12} \right)}.$$

With this weight, we obtain the weighted outcome $Y^{nw}$. Under the identifying condition, we have

$$E\left\{ Y\left( D_{1},D_{2} \right) | x_{12},x_{13} \right\}=E\left( Y^{nw} | x_{12},x_{13},z_{1},z_{2} \right), (16)$$

where regime $\left( D_{1},D_{2} \right)$ potentially and deterministically assigned $(z_{1},z_{2})$ and it could noticeably be dynamic, for instance, $D_{2}$ took the optimal dynamic regime $\hat{O}_{2}$. Taking the average with respect to ${P(x}_{12},x_{13})$, we obtain

$$E\left\{ Y\left( D_{1},D_{2} \right) \right\}=E\left( Y^{nw} | z_{1},z_{2} \right). (17)$$

Applying (17) to regimes $\left( D_{1},D_{2} \right)=\left( 1, \hat{O}_{2} \right)$ and $\left( 0, \hat{O}_{2} \right)$, we estimate the optimal blip effect $\Upsilon_{1}=E\left\{ Y\left( 1, \hat{O}_{2} \right) \right\}-E\left\{ Y\left( 0,\hat{O}_{2} \right) \right\}$, where $\hat{O}_{2}$ is the estimated optimal treating hospital. From $\hat{\Upsilon}_{1}$ , we estimate the optimal diagnosing hospital as $\hat{O}_{1}=0$. However, due to imbalance between $(z_{1},z_{2})$ and the covariates, we were not able to apply (16) to the two regimes to estimate the modification of $\Upsilon_{1}$ by age $x_{13}$, as well studied in the literature.^4-6^

Method (iii) yields the estimate $\hat{\gamma}_{1}=0.29$, which is not consistent with the medical knowledge. It also leads to the wide confidence intervals of $\gamma_{1}$and $\Upsilon_{1}$. The reason is due to imbalanced data between $(z_{1},z_{2})$ and the covariates, as well studied in the literature.^4-6^ This method does not estimate $\Gamma_{1}$, which compares between dynamic regime $(O_{1},O_{2})$ and static regime $\left( 0, 0 \right).$

- 1. **Method (iv): G-estimation based on SNMM or optimal-regime SNMM**

The G-estimation based on SNMM aims at the blip effect of diagnosing hospital $z_{1}$ while the G-estimation based on optimal-regime SNMM aims at the optimal blip effect of $z_{1}$ and the optimal diagnosing hospital $O_{1}$.^2,4,7,8^ We first study the G-estimation based on SNMM.

As described at the beginning of this section, we have already obtained the estimate $\hat{\phi}\left( x_{11},x_{13},{z_{1},x}_{2};z_{2} \right)$ for the blip effect of $z_{2}$. To estimate the blip effect $\gamma_{1}$ of large diagnosing hospital $z_{1}$, we construct the pseudo outcome by $\tilde{Y}_{1}=Y-\hat{\phi}\left( x_{11},x_{13},z_{1},x_{2};z_{2} \right)$. Under the identifying condition, with regime$\left( D_{1},D_{1} \right)=(z_{1},0)$, we have $E\left( \tilde{Y}_{1} | x_{11},x_{13},z_{1} \right)=E\{Y\left( z_{1},0 \right)|x_{11},x_{13}\}$. The model for $E\left( \tilde{Y}_{1} | x_{11},x_{13},z_{1} \right)$ is then

$$E\left( \tilde{Y}_{1} | x_{11},x_{13},z_{1} \right)=E\left( \tilde{Y}_{1} | x_{11},x_{13},0 \right)+\phi\left( x_{11},x_{13};z_{1} \right), (18)$$

where we specify a sub model for the baseline $E\left( \tilde{Y}_{1} | x_{11},x_{13},0 \right)$ and a SNMM for $\phi\left( x_{11},x_{13}; 1 \right)$ (the blip effect of $z_{1}=1$)

$$\phi\left( x_{11},x_{13}; 1 \right)=\gamma_{1,1}+\gamma_{1,2}x_{13.} (19)$$

This SNMM is the same as the first equality of SNMM (3) in Section 3.2 of the paper. Noticeably, $x_{11}$ is not significant at the significance level of 0.10. Averaging the estimate of $\phi\left( x_{11},x_{13}; 1 \right)$ over $x_{13}$, we obtain the estimate for $\gamma_{1}=\gamma_{1,1}+\gamma_{1,2}{E(x}_{13})$.

Several comments are as follows. First, it is difficult to specify a model for the baselines like $E\left( \tilde{Y}_{1} | x_{11},x_{13},0 \right)$ for a long sequence of treatments. Second, the blip effects $\phi\left( x_{11},x_{13},{z_{1},x}_{2}; 1 \right)$ and $\phi\left( x_{11},x_{13}; 1 \right)$ are estimated consecutively at two times. Therefore, it is difficult to estimate the relationship between these blip effects, for instance, the trend of the blip effects along the time. Third, to estimate SCEs under different types of regimes $\left( D_{1}, D_{2} \right)$, different versions of the G-estimation are needed, for instance, the G-estimation based on optimal-regime SNMM is developed to estimate optimal blip effects and find optimal diagnosing hospitals. Below, we describe one version of such methods, the Q-learning.^4,7,8^

As described at the beginning of this section, we have already obtained the estimate $\hat{O}_{2}=\text{arg ma}x_{z_{2}}\hat{\mu}\left( x_{11},x_{13},x_{2},z_{2} \right)$ of the optimal treating hospital $O_{2}$. From $\hat{O}_{2}$, we obtain the prediction $Y_{1}=\hat{\mu}\left( x_{11},x_{13},x_{2},\hat{O}_{2} \right)$.

For regime $D_{1}$ deterministically and potentially assigning diagnosing hospital $z_{1}$, the Q-function is $Q_{1}\left( x_{11},x_{13}; D_{1} \right)=E\{Y\left( D_{1}, \hat{O}_{2} \right)|x_{11},x_{13}\}$, where $\hat{O}_{2}$ is the estimated optimal treating hospital. Under the identifying condition, we have $Q_{1}\left( x_{11},x_{13};D_{1} \right)=E(Y_{1}|x_{11},x_{13},z_{1})$. Then the model for $E(Y_{1}|x_{11},x_{13},z_{1})$. is

$$E\left( Y_{1} | x_{11},x_{13},z_{1} \right)=E\left( Y_{1} | x_{11},x_{13},0 \right)+\varphi\left( x_{11},x_{13};z_{1} \right), (20)$$

where we specify a sub model for the baseline $E(Y_{1}|x_{11},x_{13},0)$ and an optimal-regime SNMM for $\varphi\left( x_{11},x_{13}; 1 \right)$ (the optimal blip effect of $z_{1}=1$)

$$\varphi\left( x_{11},x_{13}; 1 \right)=\Upsilon_{1,1}+\Upsilon_{1,2}x_{13.} (21)$$

Noticeably, $x_{11}$ is not significant at the significance level of 0.10. Averaging the estimate of $\varphi\left( x_{11},x_{13}; 1 \right)$ over $x_{13}$, we obtain the estimate for $\Upsilon_{1}=\Upsilon_{1,1}+\Upsilon_{1,2}{E(x}_{13})$. From the estimate $\hat{E}(Y_{1}|x_{11},x_{13},z_{1})$ of $E(Y_{1}|x_{11},x_{13},z_{1})$, we estimate the optimal diagnosing hospital by $\hat{O}_{1}=\text{arg ma}x_{z_{1}}\hat{E}(Y_{1}|x_{11},x_{13},z_{1})$. The estimated optimal diagnosing hospital is: the large one $(\hat{O}_{1}=1)$ for age < 68 years and the small one $(\hat{O}_{1}=0)$ for age $\geq$ 68 years.

Several comments are as follows. First, it is difficult to specify a model for the baselines like $E\left( Y_{1} | x_{11},x_{13},z_{1}=0 \right)$ for a long sequence of treatments. Second, the optimal blip effects are estimated consecutively at two times. Therefore, it is difficult to estimate the relationship between these optimal blip effects, for instance, the trend of the optimal blip effects along the time. Third, generally, the G-estimation does not aim to estimate SCEs under general regimes of diagnosing and treating hospitals, for instance, $\Gamma_{1}$.
